# Low Toxicity of Deoxynivalenol-3-Glucoside in Microbial Cells

**DOI:** 10.3390/toxins7010187

**Published:** 2015-01-20

**Authors:** Tadahiro Suzuki, Yumiko Iwahashi

**Affiliations:** Applied Microbiology Division, National Food Research Institute, 2-1-12 Kannon-dai, Tsukuba, Ibaraki 305-8642, Japan; E-Mail: suzut@affrc.go.jp

**Keywords:** deoxynivalenol-3-glucoside, DNA microarray, yeast, *Chlamydomonas reinhardtii*

## Abstract

Host plants excrete a glucosylation enzyme onto the plant surface that changes mycotoxins derived from fungal secondary metabolites to glucosylated products. Deoxynivalenol-3-glucoside (DON3G) is synthesized by grain uridine diphosphate-glucosyltransferase, and is found worldwide, although information on its toxicity is lacking. Here, we conducted growth tests and DNA microarray analysis to elucidate the characteristics of DON3G. The *Saccharomyces cerevisiae* *PDR5* mutant strain exposed to DON3G demonstrated similar growth to the dimethyl sulfoxide control, and DNA microarray analysis revealed limited differences. Only 10 genes were extracted, and the expression profile of stress response genes was similar to that of DON, in contrast to metabolism genes like *SER3*, which encodes 3-phosphoglycerate dehydrogenase. Growth tests with *Chlamydomonas reinhardtii* also showed a similar growth rate to the control sample. These results suggest that DON3G has extremely low toxicity to these cells, and the glucosylation of mycotoxins is a useful protective mechanism not only for host plants, but also for other species.

## 1. Introduction

**Figure 1:**
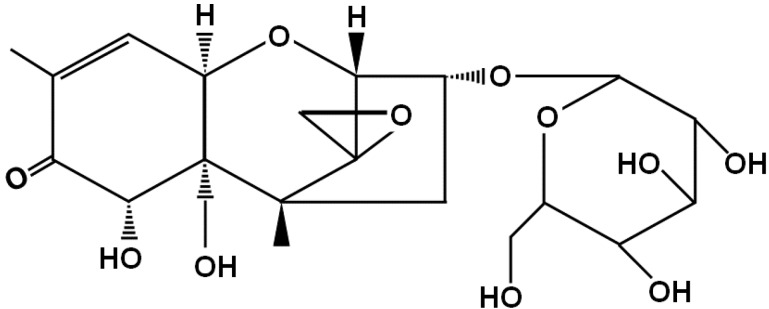
Deoxynivalenol-3-glucoside.

Disease-causing fungi like *Fusarium* species produce type B trichothecenes, and these mycotoxins contaminate grains worldwide. Because these mycotoxins or fungi cause substantial economic losses, pesticides are used to decrease fungal infection at cultivation. Plants possess resistance to fungi and mycotoxins by suppressing fungal growth and mycotoxin toxicity. To this end, some plants synthesize glycosyltransferases that metabolize secondary metabolites (plant secondary product glycosyltransferases; PSPGs) [1]. PSPGs contribute to the metabolism of pesticides or products that are secreted by microorganisms. However, the activity is strictly regulated by the specific structure. Thus, glucosyltransferase, which is a type of glycosyltransferase, has been reported to be a glucosylation enzyme of mycotoxins [2,3]. Recently, the existence of various glucosylated mycotoxins has been revealed [4]. However, at present, studies on these mycotoxins are developing; therefore, only a limited number of toxicity and detection studies have been reported. Some contamination studies on deoxynivalenol-3-glucoside (DON3G; Figure 1) have been published [5,6]. DON3G was detected at a level of up to 30 mol % in wheat, and up to 50 mol % in soybean, compared with DON [7]. This study also reported that DON3G was detected at higher levels than acetylated-DONs. Meanwhile, the same amount of DON3G compared with DON was detected in low-alcohol-content beer [8]. In that study, DON3G was also reported to be at a higher level than acetylated-DONs. Despite the contamination risk, glucosylated mycotoxin cannot be detected easily and is also not regulated; therefore it is often referred to as a masked mycotoxin.

The toxicity of DON3G varies from DON in several ways. For example, *Arabidopsis thaliana* decreases DON toxicity by expressing barley uridine diphosphate (UDP)-glucosyltransferase. In a DON exposure study with UDP-glucosyltransferase-inserted seeds, the level of DON3G significantly increased compared with the control sample in the plant extract [3]. Barley UDP-glucosyltransferase-inserted yeast cells also acquired DON resistance, and the level of DON3G increased in the culture media over time [2]. This means that regardless of the organism species, UDP-glucosyltransferase, which metabolizes DON to DON3G, influences mycotoxin resistance. Therefore, by secreting that enzyme onto the plant cell surface, feed and food products might be able to protect themselves from some, but not all, mycotoxins.

The toxicity and risk profile of DON3G is unknown. Berthiller *et al.* [9] have reported that lactic acid bacteria, such as *Enterococcus* *durans*, *Enterococcus mundtii* and *Lactobacillus plantarum*, regenerate DON from DON3G by hydrolysis. In a rat feeding study with DON3G, the recovery rates of DON3G from the urine and feces were extremely low, and more than half of the mycotoxin recovered from feces was metabolized to DON [10,11]. Also, it has been reported that the microflora in the feces metabolize DON3G to DON in several hours, and in 1 day they metabolize it to de-epoxy-DON. However, the nutrient transition time from enterocytes to mammalian cells is not always the same, and the composition of the microflora is different depending on circumstances and/or organisms. Thus, intake of DON3G has a risk of DON exposure; hence, similarly to DON, DON3G is thought to be a potential threat. However, the toxicity of DON3G has not gathered much attention. Plant and yeast studies have not elucidated sufficiently the toxic character of DON3G, though its phenotypes were well observed. Here, we evaluated the toxicity of DON3G using yeast and algae, and a comparison between type B trichothecenes was conducted to reveal their toxic character. Yeast growth and DNA microarray analysis demonstrated that DON3G has lower toxicity than DON and acetyl-DON, and the algae study suggests that DON3G has low toxicity to various species.

## 2. Results

### 2.1. Yeast Growth with DON3G

**Figure 2:**
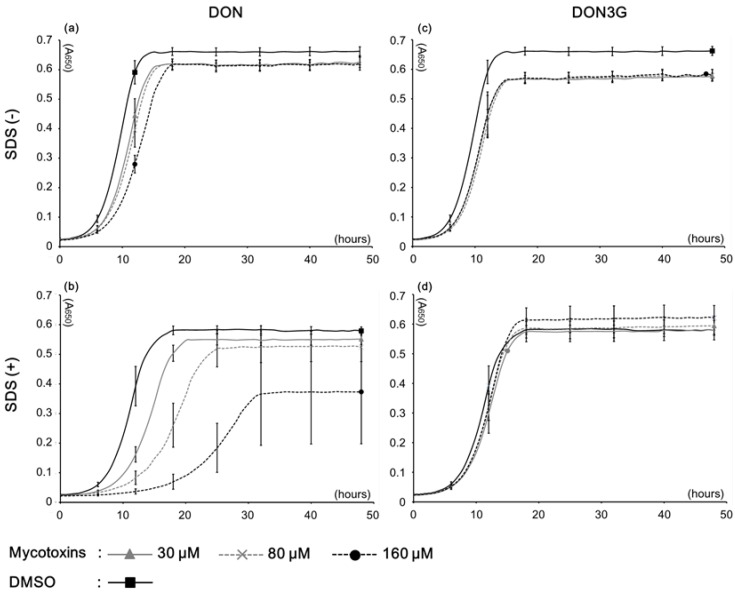
Effect of Deoxynivalenol (DON) and Deoxynivalenol-3-glucoside (DON3G) on yeast cell growth. (**a**) Growth curve under DON exposure in yeast extract peptone dextrose (YPD) medium. (**b**) Growth curve under DON exposure in YPD medium containing 0.01% SDS. (**c**) Growth curve under DON3G exposure in YPD medium. (**d**) Growth curve under DON3G exposure in YPD medium containing 0.01% sodium dodecyl sulfate (SDS). A650, absorbance at 650 nm; SDS (+): 0.01% SDS in YPD medium; Bars = SE; *n* = 3.

The yeast *PDR5* gene encodes the Pdr5 protein, which is a multidrug resistance ATP-binding cassette (ABC) transporter that localizes on the plasma membrane. Using a deletion mutant, it has been demonstrated that Pdr5 contributes to resistance against trichothecene mycotoxins [12,13]. In these studies, we used a *PDR5* mutant, Δ*pdr5*, to evaluate DON3G. Here, first, a yeast growth test was conducted. When 30–160 μM mycotoxins were used, DON at >80 μM caused growth inhibition (Figure 2a). Conversely, DON3G did not change the growth rate at any concentration, although the DON3G samples did not completely correspond with the control sample, which was treated with dimethyl sulfoxide (DMSO) (Figure 2c). Because the normal culture medium did not show an adequate difference, we next used growth media containing a low concentration of sodium dodecyl sulfate (SDS) to increase membrane permeability [14]. The growth curves with 0.01% SDS demonstrated that the DON exposure clearly caused growth inhibition compared with the normal media (Figure 2b). In contrast, even with SDS, the DON3G samples completely corresponded with each growth rate, although the control growth was marginally inhibited (Figure 2d). Inhibition rates of DON exposure samples exhibited concentration dependent growth inhibition. Conversely, DON3G did not cause any growth change, or was associated with slightly increased growth (Table 1). Regardless of the existence of SDS, DON caused significant growth inhibition (*p* < 0.05), whereas there was no growth inhibition with DON3G. The growth test results indicated that DON3G has extremely low toxicity against yeast cells.

### 2.2. DON3G Influences a Small Number of Genes

In the previous study, we conducted DNA microarray analysis to compare the toxicities of type B trichothecenes at 25 mg/kg, because the molecular weight of DON, nivalenol (NIV) and their acetylated products are relatively similar to each other. However, the molecular weight of DON3G is nearly 150% compared with the other trichothecenes used in the previous study. Therefore, we applied 80 μM DON3G, which is a concentration that caused evident growth delay under DON exposure, and used the entry to yeast cells for clustering analysis. DNA microarray with *t*-test analysis (*p* < 0.05, >1.5-fold) between DON3G exposure and the control revealed only 10 genes. *SER3*, encoding 3-phosphoglycerate dehydrogenase, which functions in the serine/glycine biosynthesis pathway, showed decreased expression. Similarly, the gene expression of *MCH2* (monocarboxylate permease-like protein), *EEB1* (acyl-coenzyme A), YMR230W-A, and an unknown protein also decreased after exposure to DON3G. The stress response genes, *ALD6*, *STP4*, *SRX1* and *ECM13*, were induced by DON3G. *HXT2* and *RGS2*, protein coding genes that are sensitive to changes in glucose level, also were induced by DON3G (Table 2).

**Table 1:** Inhibitory rate (%) and inhibitory concentration of each condition.

Medium condition	Mycotoxin	Concentrations (μM)			IC20 * (μM)
		30	80	160	
SDS (−)	DON	13.3 ± 0.64 **	14.4 ± 0.15	18.9 ± 0.08	>160
	DON3G	19.7 ± 0.98	20.2 ± 0.84	19.3 ± 2.09	- ***
SDS (+)	DON	21.1 ± 1.58	37.4 ± 8.72	71.7 ± 13.54	30.4
	DON3G	9.7 ± 3.84	7.7 ± 1.03	2.7 ± 2.45	-

* IC20: 20% inhibitory concentration of cell growth. The end time of log phase at DMSO was applied for each control of the calculation. ** ± numbers mean standard error *n* = 3. *** IC was not calculated because of unusable parameters.

**Table 2:** Significant gene expression changes revealed by DNA microarray.

Systematic No.	Gene Symbol	Fold Change	Gene Title	Description
YER081W	*SER3*	0.31	3-phosphoglycerate dehydrogenase	Serine and glycine biosynthesis
YMR230W-A	-	0.35	Putative protein of unknown function	-
YKL221W	*MCH2*	0.41	Protein with similarity to mammalian monocarboxylate permeases	Monocarboxylate transport
YPL095C	*EEB1*	0.47	Acyl-coenzymeA: ethanol O-acyltransferase	Fatty acid ethyl ester biosynthesis
YPL061W	*ALD6*	2.06	Cytosolic aldehyde dehydrogenase	Oxidative stress response
YDL048C	*STP4*	2.25	Protein containing a Kruppel-type zinc-finger domain	DNA replication stress
YKL086W	*SRX1*	2.57	Sulfiredoxin	Oxidative stress resistance DNA replication stress
YOR107W	*RGS2*	3.15	Negative regulator of glucose-induced cAMP signaling	Glucose response
YBL043W	*ECM13*	3.39	Non-essential protein of unknown function	UVA irradiation response
YMR011W	*HXT2*	5.66	High-affinity glucose transporter	Induced by low levels of glucose Repressed by high levels of glucose

### 2.3. Real-Time Polymerase Chain Reaction Analysis

**Figure 3:**
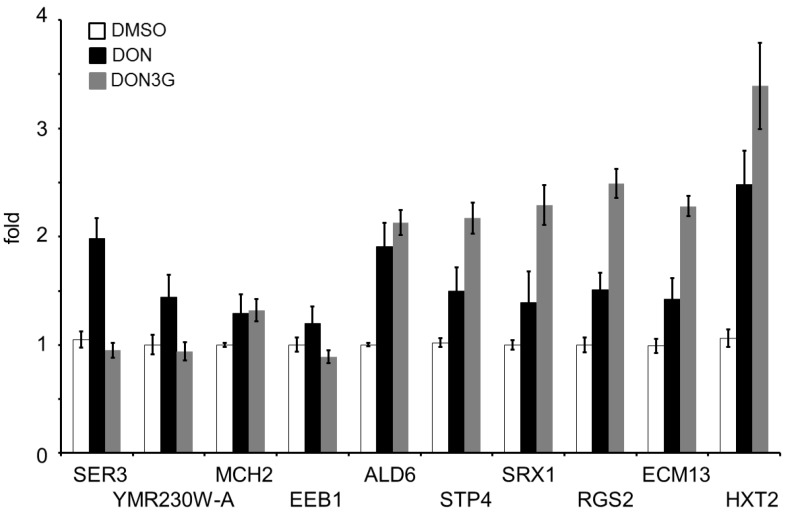
Semi-quantitative analysis of DNA microarray data. Total RNA that was used in the DNA microarray analysis and DON-treated RNA samples were prepared for synthesizing cDNA templates. Bars = SE; *n* = 3.

DNA microarray produces false-positive data that correspond to the significance level. In our study, a commercial gene chip in which approximately 5700 target gene probes were mounted was applied, and the significance level was set to 5%. Under this condition, statistical analysis permits the presence of about 280 false-positive data. Nonetheless, only 10 gene expression changes were extracted in this study. To avoid false data, we confirmed the expression profiles by semi-quantitative real-time polymerase chain reaction (PCR) analysis. As shown in Figure 3, the genes that showed decreased expression after DON3G exposure did not present significant changes compared with the control samples. In contrast, these genes were induced by exposure to DON, indicating an opposite trend, except for *MCH2*, which showed the same trend in the real-time PCR assay. *SER3* expression showed a significant difference between the DON condition and the DON3G condition. As for the DON3G-induced genes, DON induced a relatively smaller expression increase than DON3G compared with the control, and the differences were significant (unpaired *t*-test, *p* < 0.05).

### 2.4. Clustering Analysis

**Figure 4:**
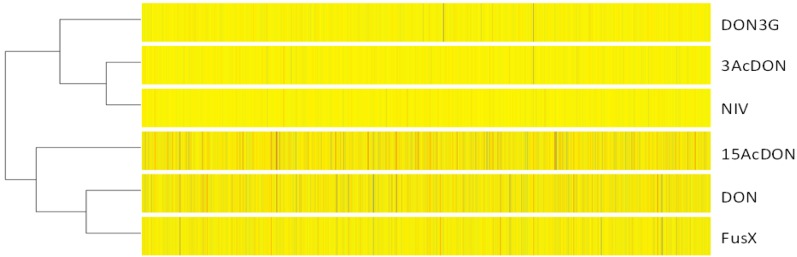
Clustering analysis of DNA microarray expression data. Except for DON3G, the gene expression data of DON, 3AcDON, 15AcDON, NIV and FusX [15] were applied by integrating the control condition samples of each condition.

In the type B trichothecene studies, we focused on DON, NIV and their acetylated products as the main contaminants rather than DON3G. These compounds were the focus of our previous study in which we obtained microarray data using *ΔPDR5* yeast cells. Therefore, we compared our previous microarray data with the current DON3G data. Because the sampling conditions were not identical, each control sample was integrated, and the clustering analysis was processed. Hierarchical clustering indicated that the trend of gene expression changes after DON3G exposure was similar to that after 3-acetyl-deoxynivalenol (3AcDON) or NIV exposure (Figure 4).

### 2.5. Growth Test with Algal Cells

**Figure 5:**
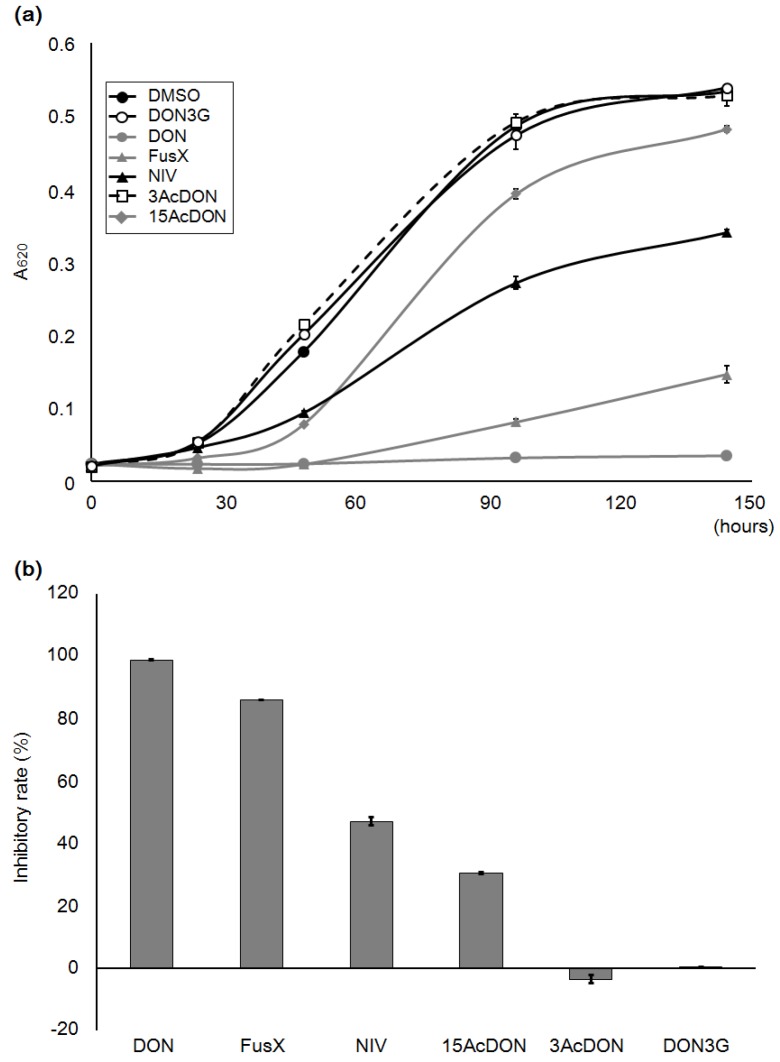
Algal cell growth test with 80 μM of mycotoxins under red light (120 μmol·m^−2^·s^−1^). The details of the photosynthetic photon flux density (PPFD; μmol·m^−2^·s^−1^) are described in the Materials and Methods. NIV, nivalenol; 3Ac-DON, 3-acetyl-DON; 15AcDON, 15-acetyl-DON; FusX, fusarenon-X (4-acetylnivalenol). (**a**) Growth curves of each test exposure. (**b**) Inhibition rates of algal growth at the end of the log phase (144 h). Bars = SE; *n* = 3.

*C. reinhardtii* is a single cell green alga that is an autotroph and a heterotroph. Hence, it is used in photosynthesis and photoresponse studies as a model cell. It has also been used as a phytotoxicity evaluation model for trichothecenes, and NIV has demonstrated moderately high toxicity in these studies [16,17]. Sensitivity to NIV has not been reported in yeast cell studies, although a number of mammalian cell studies have indicated NIV sensitivity. In our yeast cell study, DON3G also did not exhibit significant toxicity (Figure 2). However, *C. reinhardtii* was anticipated to show DON3G sensitivity, because it has differential sensitivity to some mycotoxins. Thus, we next conducted a DON3G-exposure test on *C. reinhardtii*, and examined its growth rate (Figure 5a).

The lighting intensity can be adjusted to a photosynthetic photon flux density (PPFD) of 400 to 700 nm, and at the same time the wavelength for the lighting source needs to be selected. The growth of *C. reinhardtii* does not show a significant difference between the different wavelength conditions, although the red light (660 nm) contributes to the most efficient growth [17]. In this study, therefore, we applied red lighting. DON, acetylated DON (3-acetyl-DON; 3AcDON, 15-acetyl-DON; 15Ac-DON), NIV and FusX exposures were examined, as well as DON3G. The cell growth with 80 μM DON3G showed a similar curve to that of the DMSO control sample. All the other compounds, except 3AcDON, demonstrated reduced growth, whereas DON exposure caused complete growth inhibition (Figure 5a). When cell growth was examined at the end of the log phase, DON and FusX exposure caused significant growth inhibition while NIV and 15AcDON exposure showed a moderate level of inhibition (unpaired *t*-test, *p* < 0.01, Figure 5b). Conversely, DON3G and 3AcDON did not cause cell growth inhibition.

## 3. Discussion

Mycotoxins are secondary metabolites produced by pathogenic fungi. They are often glucosylated by an enzymatic reaction with β-glucosyl-transferase, which is excreted by the host plant onto the plant surface. When glycosylated, mycotoxins exhibit considerably less toxicity; therefore, mycotoxin glucosylation is clearly a host plant resistance response. If a mycotoxin causes cellular damage, it is natural that the host plant would evolve a mechanism for decreasing the toxicity. For example, it has been identified in plant studies that glucosylated DON is less toxic than DON [2,3]. However, it is unclear whether the attenuation in toxicity by glucosylation is a plant cell-specific character, because a sufficient amount of refined glucosylation product is difficult to obtain for toxicity studies. DON3G has been synthesized by an organic chemical technique [5,18], and by a molecular biological technique, which is inserted into microbes or plants [19]. However, its availability for study is still lower than other mycotoxins; hence, this research is useful for understanding the features of DON3G.

In our previous study, we examined gene expression changes after exposure to DON, NIV and their acetylated products [15]. Therefore, in this study, 80 μM, which was easy to compare with the other data, was used for growth tests and DNA microarray analysis. Additionally, 160 μM DON3G was applied to elucidate the influence of concentration change on growth. However, DON3G did not cause significant growth differences, as opposed to those caused by DON, especially with the addition of 0.01% SDS to the medium. It is thought that *S. cerevisiae* Pdr5 is the transporter for trichothecenes like DON and T-2 toxin. In contrast, 3AcDON, which is the less toxic product to various cell lines, and NIV, which shows high toxicity to mammalian cells, are not affected by Pdr5 deletion. It is likely that yeast cells have a number of transporters for stress resistance. Under a multiple transporter scenario, it is possible that deletion of Pdr5 would not affect DON3G.

The real-time PCR data of *SER3* did not indicate a significant difference between the control and DON3G treatment, whereas the DNA microarray data did, demonstrating a slight decrease; thus, it seems that *SER3* expression may not have changed in response to DON3G exposure as expected from the microarray result. On the other hand, *SER3* was significantly induced by DON exposure; hence, the gene expression patterns after DON and DON3G exposure were different. YMR230W-A and *EEB1* had a similar expression trends; however, the differences between the treatments were lower than that of *SER3*. The other tested genes showed similar expression patterns between DON and DON3G, although the expression levels were different. Taken together, the gene expression pattern of *SER3* may be useful for discriminating the toxicity differences between DON and DON3G. 3-Phospho-glycerate dehydrogenase, encoded by *SER3*, plays a role in the metabolism of 3-phospho-glycerate derived from the glycolytic system. As the gene expression profile of the glycolytic system changes as a result of mycotoxin exposure [20], the change in expression of *SER3* may be attributable to that influence. However, the induction level of *HXT2*, which encodes a glucose transporter induced by low glucose conditions, was higher by DON3G than by DON. Generally, it is thought that the DON-induced gene expression change is larger than that of DON3G; hence, the gene expression pattern of *HXT2* is also valuable. Most of the detected genes do not encode proteins that localize in mitochondria. Inhibition of mitochondrial function causes cell death, which is easy to observe at lethal toxic agent exposures. In fact, highly toxic type B trichothecenes like DON decrease the expression of mitochondria ribosomal genes [15]. Meanwhile, less toxic mycotoxins like 3AcDON or NIV do not cause such radical expression patterns. Taken together, the data show that DON3G does not affect critical metabolic functions. Robust function of mitochondria might be a key point of the more highly induced genes after DON3G exposure compared with that of DON. However, more detailed information is necessary to elucidate the results of reverse-transcription PCR. A DON trap model by the yeast cell wall layer has been reported [21]. Moreover, DON3G has a larger molecular weight and a different structure than DON. Therefore, it is possible that the trap efficiency is different. However, we cannot exclude the possibility that DON3G affects intercellular components, because DNA stress response genes were induced, and cellular metabolism genes like *SER3* were repressed. Nevertheless, the stress response was significantly limited, suggesting only a marginal strain. Thus, the role of these genes in this study remains unresolved. Clarifying their role will elucidate the cellular response mechanism to the toxicity of DON3G. When compared with other type B trichothecene mycotoxins, DON3G belonged to the low toxicity cluster, which includes 3AcDON and NIV, suggesting that DON3G has a different character from the highly toxic mycotoxins DON, 15AcDON and FusX. Taken together, these results indicate that the toxicity of DON3G to yeast cells is low.

The toxicity of DON3G to *C. reinhardtii* was also not significant. *C. reinhardtii* is more sensitive to type B trichothecene mycotoxins than yeast; especially NIV, which caused significant growth inhibition in the toxicity study. This character is similar to that of mammalian cell lines. Moreover, *C. reinhardtii* is a model cell for photo reactions or photosynthesis [22]. Additionally, *C. reinhardtii* is sensitive to continuously high intensity of light, fast water flow, and somewhat high concentrations of DMSO [23]. Regardless, the toxicity to *C. reinhardtii* corresponded to that of yeast. It has been reported that glucosylation of mycotoxins contributes to the attenuation of toxicity [3]. Therefore, considering the results of this study, it appears that the attenuation effect is not exclusive to host plants.

Finally, there are some concerns. Barley UDP-glucosyltransferase-introduced *A. thaliana* increases the resistance to DON [3]. On the other hand, when measuring lethality (LD50) using *A. thaliana* leaf, the toxicity of both NIV and FusX was low, while DON and 3AcDON showed a similar level of toxicity [24]. Many studies using mammalian or yeast cells indicated a difference between DON and 3AcDON, and yeast cells were sensitive to FusX but not to NIV. Conversely, NIV sensitivity has been reported in a study using mammalian cells. It is apparent that sensitivity to mycotoxins depends on the species. Therefore, toxicity evaluation of glucosylated mycotoxins to different species and cell lines is even more necessary.

There are several recent reports regarding glucosylated and other masked mycotoxins [25,26,27]. It is thought that the stress response of the host plant functions to attenuate the toxicity of various mycotoxins. Mycotoxin-derived cellular lesions are important for fungal invasion of host plants; hence, glucosylation may well be one component of the plant’s antifungal resistance arsenal. Because of their abundance and potential risk, glucosylated mycotoxins will be the subject of increased study in the near future.

## 4. Materials and Methods

### 4.1. Chemicals

Commercial products of the mycotoxins, deoxynivalenol (DON; Santa Cruz, Dallas, TX, USA), NIV (Wako, Osaka, Japan), 3AcDON (Sigma, St. Louis, MO, USA), 15AcDON (Santa Cruz), FusX (Sigma) and DON3G (Wako) were dissolved in DMSO (Wako) to prepare sample solutions.

### 4.2. Biomaterials

A glycerol stock of yeast *Saccharomyces cerevisiae* Δ*pdr5*—plasma membrane ABC transporter Pdr5 deletion mutant strain of *S. cerevisiae* BY4743 (MATa/α his3Δ1/his3Δ1 leu2Δ0/leu2Δ0 LYS2/lys2Δ0 met15Δ0/MET15 ura3Δ0/ura3Δ0; Open Biosystems, Huntsville, AL, USA)—was thawed, and cells were transferred by an inoculation needle into 5 mL of YPD medium (1% yeast extract, 2% peptone, and 2% dextrose) in glass tubes. Triplet samples were pre-incubated with 150 rpm rotation at 25°C for 2 days. *Chlamydomonas reinhardtii* wild-type strain 137C was provided by Prof. M. Tsuzuki (Department of Applied Life Science, Tokyo University of Pharmacy and Life Science, Tokyo, Japan). Algal cells were picked from a slant culture on Tris-acetate-phosphate (TAP) medium agar [28] and were inoculated into 100 mL of TAP medium. Culture media were pre-incubated with 100 rpm rotation at 25°C, and constant lighting for at least 3 days.

### 4.3. Yeast Growth

Pre-incubated *S. cerevisiae PDR5* mutant was diluted in YPD medium. At approximately OD_650_ = 0.01, the cell culture containing DON, DON3G or DMSO was dispensed into a 96-well plate. Each volume of mycotoxin was adjusted by adding DMSO. SDS was also added into culture media, and test plates were incubated at 25 °C. Optical density, which indicates growth rate, was measured by a plate reader (Filter Max F5, Molecular Devices, Sunnyvale, CA, USA). For measuring the inhibition rate of cell growth, each sum of area under the growth curve was calculated until the time that corresponded to the end of the log phase of growth of the control sample (36 h), and it was divided by that of DMSO data, which was set as a control. Inhibition rates were tested by unpaired *t*-test analysis (*p* < 0.05) compared with DMSO control, respectively. Twenty percent of inhibition concentration (IC20) was calculated by using asymptotic line that was derived from time dependent inhibition rates.

### 4.4. DNA Microarray Analysis

Pre-incubated cultures were diluted to OD_650_ of about 0.85–1.0. Cultures were centrifuged at 1580× *g* for 5 min, and cell pellets were prepared for the RNA study. Total RNA was prepared using a commercial kit (FastRNA Pro Red kit, MP Biomedicals, Irvine, CA, USA) following the supplier’s instructions. Contaminated genomic DNA was removed by RNeasy mini kit (Qiagen, Venlo, The Netherlands). The quality of the total RNA was evaluated with a nucleic acid analyzer (Experion, Bio-Rad, Hercules, CA, USA). RNA samples were used for synthesizing labeled RNA with 3’IVT Express kit, and DNA microarrays (GeneChip Yeast Genome 2.0 array) were processed according to the manufacturer’s instructions (Affymetrix, Santa Clara, CA, USA). The DNA microarray data were transferred into GeneSpring analysis software (ver. 12, Agilent Technologies, Santa Clara, CA, USA). Cluster analyses were performed for each condition. After using the MAS5 algorithm to obtain summarized probeset-level expression data, the average expression of triplicates was normalized to the control condition. An unpaired *t*-test was used for statistical analysis, and significant differences in gene expression were selected using a *p* value < 0.05. To avoid detection of false positives, a multiple testing correction (Benjamini-Hochberg FDR) was applied to obtain corrected *p* values. Each character of a selected gene was confirmed according to the Saccharomyces Genome Database (SGD; http://www.yeastgenome.org/). The microarray data set has been assigned the accession number, GSE63663, in the Gene Expression Omnibus Database (GEO; http://www.ncbi.nlm.nih.gov/geo/).

### 4.5. Real-Time PCR Analysis

One microgram of genomic DNA-free total RNA, which was used for the microarray analysis, was reverse transcribed (PrimeScript first cDNA synthesis kit, Takara Bio, Shiga, Japan). Similarly treated DON-exposed samples were also prepared. First strand cDNA was diluted five times with Tris-EDTA, pH 8.0. Each 1 μL of cDNA solution was used as a DNA template. Primer sets were designed on primer3 (Table 3). The primer set of *ACT1*, which encodes the actin protein, was used for internal control, and the *PDR5* primer set was used for negative control. The *ACT1* primer set, which has been previously reported [29], was prepared. The same volume of mycotoxin-treated sample templates was also dispensed on the plate. A total of 19 μL of reaction mix [0.4 μL of 10 μM primer each, 8.2 μL of distilled water, 10 μL of 2× Master mix (KAPA SYBR FAST qPCR kit, Kapa Biosystems, Woburn, MA, USA)] was added into a PCR plate. Sample plates were processed at 95 °C for 2 min, followed by 40 cycles of 95 °C for 3 s and 60 °C for 20 s in a thermal cycler (MX3000P, Agilent Technologies, Santa Clara, CA, USA). The amplified *ACT1* product was used as an internal control, and each triplicate was averaged. Unpaired *t*-test analyses were conducted between control and DON3G samples (*p* < 0.05). One-way analysis of variance analyses were also conducted between control, DON, and DON3G samples (*p* < 0.05) to consider gene expressions of DON.

### 4.6. C. reinhardtii Growth

Light-emitting diode (LED) conditions were manually constructed on an LED platform (SPL-100-CC; Revox, Kanagawa, Japan) with red (660 nm) diodes, and the photon flux density was modulated by a pulse-width modulation dimmer controller. The spectrum of LED irradiation was measured by an illuminance spectrophotometer (CL-500A; Konica Minolta, Tokyo, Japan) as irradiances (W/m^2^). The total irradiances of spectra from 400 to 700 nm were counted and the PPFD of each spectrum condition was calculated with the following formula: PPFD (μmol·m^−2^·s^−1^) = [irradiance (W/m^2^) × spectrum (m) × 10^−9^]/[Planck’s constant (6.626 × 10^−34^; J.s) × speed of light (2.998 × 10^8^; m/s) × Avogadro constant (6.022×10^23^; mol^−1^)] × 10^6^. Mycotoxins were added into TAP medium at a final concentration of 80 μM. The same volume of DMSO was added as a control. For measuring the inhibition rate of cell growth, each sum of area under the growth curve was calculated until the time that corresponded to the end of the log phase of growth of the control sample (144 h), and it was divided by that of DMSO data, which was set as a control. Inhibition rates were tested by unpaired *t*-test analysis (*p* < 0.05) compared with DMSO control, respectively.

**Table 3:** Primer sets for real-time PCR.

Gene symbol	Direction	Sequence	Product size (bp)
*SER3*	forward	TCACCAAAATGTACCAGGTGT	227
	reverse	TGACAGTATGAGAATCGATTGCA	
*YMR230W-A*	forward	GGATGTGTTACGATGCAGACA	170
	reverse	ATATGGCGCGTTCTTGAAGG	
*MCH2*	forward	CGTGGGTTTTGCGTACTTTG	194
	reverse	GTGACCTTTAGACTCTCCTAGGT	
*EEB1*	forward	TCCAGTTACAGGTGAAAACGT	164
	reverse	ACTCATCAAAGCTGCCCAAG	
*ALD6*	forward	AGATGTTGAAGGCCGGTACC	185
	reverse	TGACGGAAAGAAATGCAGGT	
*STP4*	forward	TTTGCATTTCGAGTACCCGC	183
	reverse	TGTGTGTATGTATGAGTCGGTG	
*SRX1*	forward	CTGGGCGTGCGAGTCAAG	176
	reverse	ATGTCGAGACTGCTGCCC	
*RGS2*	forward	AGGAATTCTCAACTCGGGGA	173
	reverse	TCCACAGATGATGAAGAGGCT	
*ECM13*	forward	CGAGCAGACGGATGAACTTG	214
	reverse	TACGGAACCATCGTCGACAT	
*HXT2*	forward	GGGTATGTCTTCATGGGCTGT	180
	reverse	TATAATCTCTTATTCCTCGGAAACTC	
*PDR5*	forward	ACAGTGAGAGATGGAGAAATTATGG	170
	reverse	GTCCATCTTGGTAAGTTTCTTTTCTT	
*ACT1*	forward	ATTGCCGAAAGAATGCAAAAGG	220
	reverse	CGCACAAAAGCAGAGATTAGAAACA	
